# Association of easy albumin-bilirubin score with increased mortality in adult trauma patients

**DOI:** 10.3389/fsurg.2024.1280617

**Published:** 2024-04-24

**Authors:** Shiun-Yuan Hsu, Cheng-Shyuan Rau, Ching-Hua Tsai, Sheng-En Chou, Wei-Ti Su, Ching-Hua Hsieh

**Affiliations:** ^1^ Department of Trauma Surgery, Kaohsiung Chang Gung Memorial Hospital and Chang Gung University College of Medicine, Kaohsiung, Taiwan; ^2^ Department of Neurosurgery, Kaohsiung Chang Gung Memorial Hospital and Chang Gung University College of Medicine, Kaohsiung, Taiwan

**Keywords:** albumin-bilirubin (ALBI), easy albumin-bilirubin (EZ-ALBI), liver function, mortality, trauma

## Abstract

**Introduction:**

The easy albumin-bilirubin (EZ-ALBI) score is calculated using the equation: total bilirubin (mg/dl) − 9 × albumin (g/dl), and is used to evaluate liver functional reserve. This study was designed to investigate whether the EZ-ALBI score serves as an independent risk factor for mortality and is useful for stratifying the mortality risk in adult trauma patients.

**Methods:**

We retrospectively reviewed data from the registered trauma database of the hospital and included 3,637 adult trauma patients (1,241 deaths and 2,396 survivors) due to all trauma caused between January 1, 2009, and December 31, 2021. The patients were allocated to the two study groups based on the best EZ-ALBI cutoff point (EZ-ALBI = −28.5), which was determined based on the area under the receiver operating characteristic curve.

**Results:**

Results revealed that the non-survivors had a significantly higher EZ-ALBI score than the survivors (−26.4 ± 6.5 vs. −31.5 ± 6.2, *p* < 0.001). Multivariate logistic regression analysis revealed that EZ-ALBI ≥ −28.5was an independent risk factor for mortality (odds ratio, 2.31; 95% confidence interval, 1.63–3.28; *p* < 0.001). Patients with an EZ-ALBI score ≥ −28.5 presented with 2.47-fold higher adjusted mortality rates than patients with an EZ-ALBI score < −28.5. A propensity score-matched pair cohort of 1,236 patients was developed to reduce baseline disparities in trauma mechanisms. The analysis showed that patients with an EZ-ALBI score ≥ −28.5 had a 4.12 times higher mortality rate compared to patients with an EZ-ALBI score < −28.5.

**Conclusion:**

The EZ-ALBI score was a significant independent risk factor for mortality and can serve as a valuable tool for stratifying mortality risk in adult trauma patients by all trauma causes.

## Introduction

Because liver function is perceived as a competing issue related to patient mortality, the assessment of the liver function reserve is particularly important in the clinical setting (1). In addition to the traditional evaluation tools of the model for end-stage liver disease (MELD) score (2) or the Child-Turcotte-Pugh (CTP) classification (3), an alternative measure of liver function based solely on albumin and bilirubin, the albumin-bilirubin (ALBI) score, was proposed in international collaboration as a simple and objective method for the assessment of liver function in patients with hepatocellular carcinoma (4). Since its introduction, the ALBI score has been validated by several research groups to predict the outcome of patients with resectable or locally advanced hepatoma (5–9) as well as in those with advanced hepatoma receiving local or systemic therapy (6, 8–12). Additionally, it serves as an important biomarker for liver disease progression to reflect the possibility of hepatic failure and liver-related mortality (13–19). ALBI grade is also useful as a prognostic factor in patients with cholangiocarcinoma (20), intrahepatic cholangiocarcinoma (21), colorectal cancer with liver metastases (22), pancreatic cancer with liver metastases (23), and primary biliary cholangitis (24). Furthermore, a strong association between ALBI and mortality has been identified in many non-hepatological conditions, such as gastric cancer (25), lung cancer (16, 26, 27),esophageal cancer (28), glioma (29), medulloblastoma (30), heart failure (31, 32), acute pancreatitis (33), and aortic dissection (34).

The ALBI score is calculated using the following formula: [(log10 bilirubin (μmol/L) × 0.66) + [albumin (g/L) × −0.0852]. The complexity of the calculation of the ALBI score limits its applicability. Therefore, an easy-ALBI (EZ-ALBI) score was recently developed to replace the ALBI score based on the regression coefficients of serum albumin and bilirubin levels using a multivariate Cox proportional hazards model, and calculated by the equation: total bilirubin (mg/dl) − 9 × albumin (g/dl) (35). The EZ-ALBI score showed a high linear correlation (correlation coefficient, 0.965; *p* < 0.001) with the ALBI score in the entire cohort and different subgroups of patients with hepatoma (36). With easy calculation and a more user-friendly assessment, the EZ-ALBI score can evaluate liver functional reserve in patients with liver diseases receiving various treatment modalities (35–39).

While the liver plays a significant role in producing albumin, there are several other factors that can impact the albumin levels in the body, especially in the context of trauma patients, including increased capillary permeability and fluid shifts, inflammatory response, malnutrition, and impaired renal function (40–47). In addition, the level of bilirubin, a breakdown product of hemoglobin from red blood cells and primarily processed and excreted by the liver, may be influenced not only by liver function but also by hemolysis and processing of cell-free hemoglobin from the circulation (48–50). Under the hypothesis that the EZ-ALBI score may be associated with the mortality risk of trauma patients, this study aimed to investigate whether the EZ-ALBI score serves as an independent risk factor for mortality and is useful for stratifying the mortality risk of adult patients with all trauma causes. In this study, the primary outcome was the in-hospital mortality rate.

## Materials and methods

### Ethics statement

The study was conducted in accordance with the Declaration of Helsinki, and approved by the Institutional Review Board of Chang Gung Memorial Hospital (protocol code 202201380B0 and date of approval 2022/09/15). The need for informed consent was waived according to the IRB regulations because of its retrospective study of design.

### Study population and data collection

**Figure 1:**
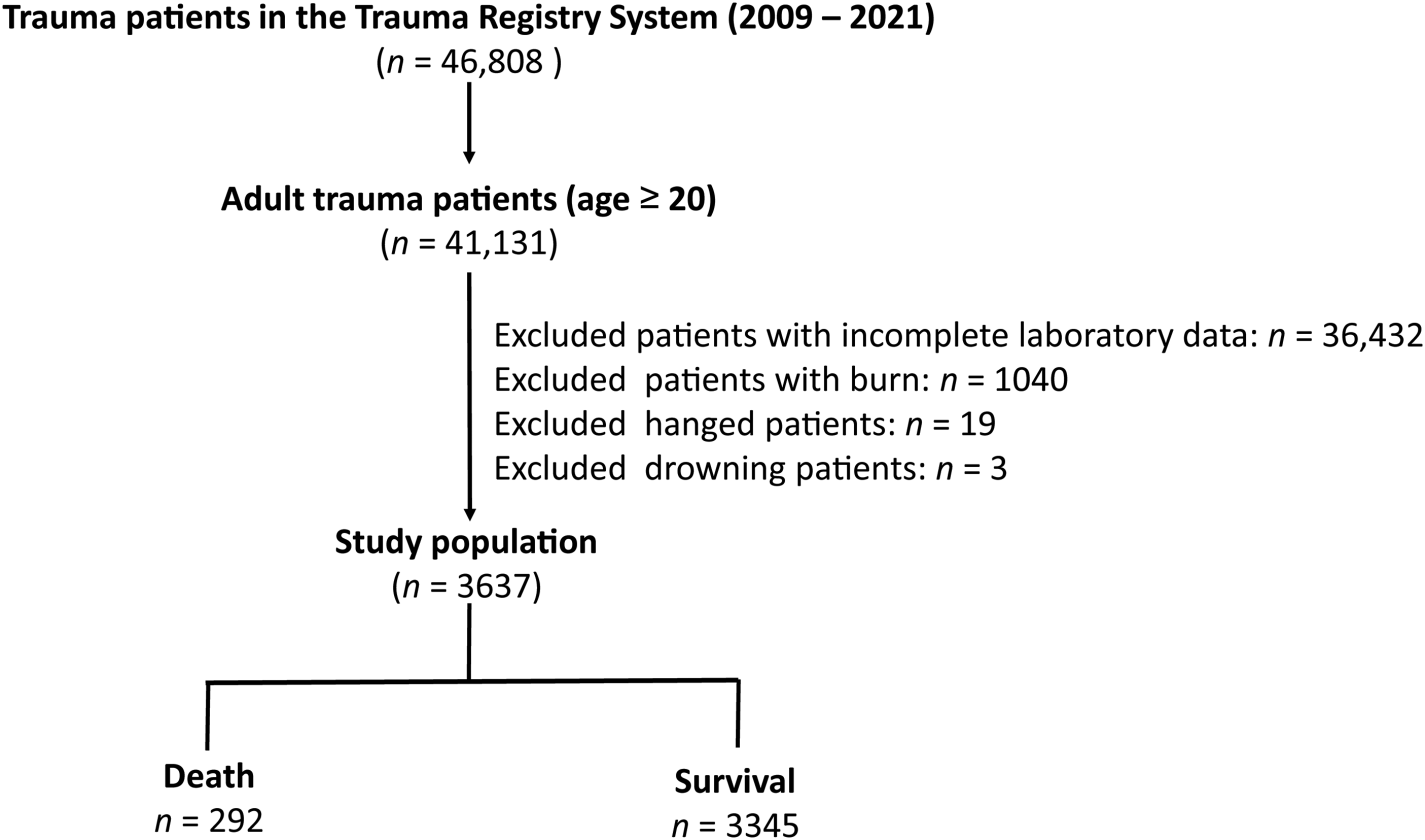
Flowchart illustrating the inclusion of hospitalized adult trauma patients by all trauma causes from the registered trauma database. Those patients who lacked the albumin or bilirubin data, who had burn injury, hang injuries, and drown, were excluded from the study population. The study population were assigned into death and survival two groups.

There were 46,808 hospitalized patients injured by all trauma causes in the Trauma Registry System of the Chang Gung Memorial Hospital between January 1, 2009, and December 31, 2021 (51–54) (Figure 1). Of the 41,131 adult patients aged ≥20 years, after excluding patients who lacked data on albumin or bilirubin (*n* = 36,432), those with burn injuries (*n* = 1,040), hanging injuries (*n* = 19), and patients who drowned (*n* = 3), 3,637 adult trauma patients were included in the study population. The major liver injuries indicated that the trauma patients had suffered an abbreviated injury scale (AIS) ≥ 3 liver injury in the abdomen. We retrieved the medical information of the study population from a registered trauma database. The data included sex, age, levels at admission of serum albumin, total bilirubin, glucose, white blood cells count, hemoglobin (Hb), hematocrit (Hct), platelets, aspartate aminotransferase (AST), alanine aminotransferase (ALT), blood urea nitrogen (BUN), and creatinine, trauma regions, trauma mechanism, pre-existing comorbidities, a Glasgow Coma Scale (GCS) score, an Injury Severity Score (ISS), hospital length of stay (LOS), and in-hospital mortality. The EZ-ALBI score was calculated according to the equation: total bilirubin (mg/dl) − 9 × albumin (g/dl).

### Statistical analyses

Categorical data were compared using a two-sided Fisher's exact test. Normally distributed continuous data were estimated using the Kolmogorov–Smirnov test. Non-normally distributed continuous data were analyzed using the Mann–Whitney *U*-test, and continuous data with a normal distribution were compared using analysis of variance with Bonferroni *post hoc* correction. Continuous data are expressed as the mean ± standard deviation. Non-normal distributed continuous data are presented as medians with interquartile ranges (IQR) between Q1 and Q3. Multivariate logistic regression was used to analyze the univariate predictive variables, resulting in patient mortality and identify independent risk factors for mortality. The predictive performance of EZ-ALBI for patient mortality was determined based on the area under the curve (AUC) of the receiver operating characteristic curve (ROC). Based on a value determined using sensitivity + specificity − 1, the maximal Youden index, the best cutoff point was derived from ROC. In addition to the comparison between the death and survival groups of patients, a further comparison of the patients allocated into two groups based on the best cutoff point of the EZ-ALBI value was performed with the presentation of an adjusted odds ratio (AOR) of mortality with 95% confidence intervals (CIs), calculated using logistic regression under the control of variables with significant differences in patient injury characteristics. To effectively account for any initial differences in baseline characteristics among patient groups divided by the best cutoff point of the EZ-ALBI value, particularly the impact of different trauma mechanisms, a cohort with a 1:1 propensity score matching was created using the Greedy strategy with a caliper width of 0.2 and the NCSS 10 software (NCSS Statistical program, Kaysville, Utah, USA). The statistical analyses were performed using SPSS Statistics (version 23.0; IBM Corp., Armonk, NY, USA). Statistical significance was set at *p* < 0.05.

## Results

### Injury and patient characteristics

A comparison between 292 deceased and 3,345 surviving patients revealed that the non-survivors comprised significantly more males and were older than the surviving patients (Table 1). Patients who died had a significantly higher EZ-ALBI score than those who survived (−26.4 ± 6.5 vs. −31.5 ± 6.2, *p* < 0.001). The non-survivors had a significantly lower serum albumin level than the survivors (3.1 ± 0.8 vs. 3.6 ± 0.7, *p* < 0.001), while there was no significant difference in total bilirubin level between these two groups of patients (1.1 ± 0.9 vs. 1.0 ± 1.3, *p* = 0.173). The non-survivors had a significantly different level of glucose, AST, ALT, BUN, Cr than the survivors. The non-survivors had more incidences of AIS ≥ 3 injuries to head/neck and external body regions but fewer AIS ≥ 3 injuries to extremities than the survivors. Regarding comorbidities, significantly higher rates of pre-existing comorbidities of coronary artery disease (CAD), end-stage renal disease (ESRD), and liver cirrhosis were found in patients who died than in those who survived. Patients who died presented with a significantly lower GCS (median [IQR, Q1–Q3], GCS: 7 ([3–15]) vs. 15 ([13–15]), *p* < 0.001) but a higher ISS (25 ([16–29]) vs. 12 ([9–20]), *p* < 0.001) than patients who survived. Patients who died had a significantly shorter hospitalization period than those who survived (14.2 vs. 17.7 days, *p* = 0.001).

**Table 1:** Comparison of the injuries and patient characteristics of death and survival patients in the study population.

Variables	Death *n* = 292	Survival *n* = 3,345	OR (95%CI)	*p*
Gender				0.001
Male, *n* (%)	204 (69.9)	2,018 (60.3)	1.52 (1.18–1.98)	
Female, *n* (%)	88 (30.1)	1,327 (39.7)	0.66 (0.51–0.85)	
Age, years (SD)	60.8 ± 19.2	56.8 ± 19.5	–	0.001
EZ-ALBI	−26.4 ± 6.5	−31.5 ± 6.2	–	<0.001
Albumin (g/dl)	3.1 ± 0.8	3.6 ± 0.7	–	<0.001
Total-bilirubin (mg/dl)	1.1 ± 0.9	1.0 ± 1.3	–	0.173
Glucose (mg/dl)	215.8 ± 98.3	170.2 ± 82.6	–	<0.001
White blood cells (×10^3^)	12.5 ± 6.2	12.1 ± 8.7	–	0.441
Hb (g/dl)	12.6 ± 2.5	12.9 ± 2.6	–	0.077
Hct (%)	37.8 ± 6.9	38.5 ± 6.0	–	0.068
Platelets (10^3^/ul)	214.2 ± 69.6	221.8 ± 80.4	–	0.128
AST (U/L)	169.1 ± 600.0	99.5 ± 194.8	–	<0.001
ALT (U/L)	86.5 ± 271.0	65.1 ± 115.5	–	0.011
BUN (mg/dl)	23.8 ± 20.2	18.1 ± 14.2	–	<0.001
Cr (mg/dl)	2.0 ± 2.3	1.3 ± 1.8	–	<0.001
Trauma regions (AIS ≥ 3)				
Head/neck, *n* (%)	214 (73.3)	1,148 (34.3)	5.25 (4.01–6.87)	<0.001
Face, *n* (%)	2 (0.7)	21 (0.6)	1.09 (0.26–4.68)	0.906
Thoracic, *n* (%)	72 (24.7)	683 (20.4)	1.28 (0.97–1.69)	0.087
Abdomen, *n* (%)	34 (11.6)	376 (11.2)	1.04 (0.72–1.51)	0.835
Extremities, *n* (%)	58 (19.9)	1,005 (30.0)	0.58 (0.43–0.78)	<0.001
External, *n* (%)	5 (1.7)	9 (0.3)	6.46 (2.15–19.40)	<0.001
Comorbidities				
CVA, *n* (%)	16 (5.5)	175 (5.2)	1.05 (0.62–1.78)	0.856
HTN, *n* (%)	106 (36.3)	1,142 (34.1)	1.10 (0.86–1.41)	0.456
CAD, *n* (%)	31 (10.6)	200 (6.0)	1.87 (1.25–2.78)	0.002
CHF, *n* (%)	5 (1.7)	35 (1.0)	1.65 (0.64–4.24)	0.295
DM, *n* (%)	67 (22.9)	649 (19.4)	1.24 (0.93–1.65)	0.144
ESRD, *n* (%)	26 (8.9)	100 (3.0)	3.17 (2.02–4.97)	<0.001
Liver cirrhosis, *n* (%)	23 (7.9)	122 (3.6)	2.26 (1.42–3.59)	<0.001
GCS, median (IQR)	7 (3–15)	15 (13–15)	–	<0.001
ISS, median (IQR)	25 (16–29)	12 (9–20)	–	<0.001
1–15, *n* (%)	54 (18.5)	1,919 (57.4)	0.17 (0.12–0.23)	<0.001
16–24, *n* (%)	66 (22.6)	867 (25.9)	0.84 (0.63–1.11)	0.213
≥25, *n* (%)	172 (58.9)	559 (16.7)	7.14 (5.56–9.17)	<0.001
Time to Death, days (SD)	14.2 ± 16.5			0.001
Hospital LOS, days (SD)		17.7 ± 16.4	–	

AIS, abbreviated injury scale; ALT, alanine aminotransferase; AST, aspartate aminotransferase; BUN, blood urea nitrogen; CAD, coronary artery disease; CHF, congestive heart failure; CI, confidence interval; Cr, creatinine; CVA, cerebral vascular accident; DM, diabetes mellitus; EZ-ALBI, easy albumin-bilirubin; ESRD, end-stage renal disease; GCS, Glasgow Coma Scale; Hb, hemoglobin; Hct, hematocrit; HTN, hypertension; IQR, interquartile range; ISS, injury severity score; LOS, length of stay; OR, odds ratio; SD, standard deviation.

A comparison of the injuries and patient characteristics of the patients with and without major liver injuries revealed the patients with major liver injuries were significantly younger, had fewer incidences of HTN and DM, sustained significantly more severe injuries, and stayed longer in the hospital than those without major liver injuries (Table 2). However, these two groups of patients with or without major liver injury did not present significant differences in the level of albumin, total bilirubin, or the derived value of EZ-ALBI. The mortality and adjusted mortality corrected by age, incidences of HTN and DM, and ISS between the patients with and without major liver injuries did not present significant differences.

**Table 2:** Comparison of the injuries and patient characteristics of the trauma patients with and without major liver injury.

Variables	Major liver injury *n* = 153	No major liver injury *n* = 3,484	OR (95%CI)	*p*
Male, *n* (%)	88 (57.5)	2,134 (61.3)	0.86 (0.62–1.19)	0.354
Age, years (SD)	43.8 ± 17.4	57.7 ± 19.4	–	<0.001
EZ-ALBI	−30.1 ± 5.8	−31.1 ± 6.4	–	0.060
Albumin (g/dl)	3.5 ± 0.7	3.6 ± 0.7	–	0.170
Total-bilirubin (mg/dl)	1.0 ± 0.7	1.1 ± 1.3	–	0.761
Comorbidities				
CVA, *n* (%)	1 (0.7)	190 (5.5)	0.11 (0.02–0.82)	0.009
HTN, *n* (%)	22 (14.4)	1,226 (35.2)	0.31 (0.20–0.49)	<0.001
CAD, *n* (%)	6 (3.9)	225 (6.5)	0.59 (0.26–1.35)	0.208
CHF, *n* (%)	0 (0.0)	40 (1.1)	–	0.183
DM, *n* (%)	17 (11.1)	699 (20.1)	0.50 (0.30–0.83)	0.006
ESRD, *n* (%)	1 (0.7)	125 (3.6)	0.18 (0.03–1.27)	0.052
GCS, median (IQR)	15 (13–15)	15 (12–15)	–	0.946
ISS, median (IQR)	21 (14–31)	13 (9–20)	–	<0.001
Hospital LOS, days (SD)	20.2 ± 18.8	17.3 ± 16.3	–	0.032
Mortality, *n* (%)	14 (9.2)	278 (8.0)	1.16 (0.66–2.04)	0.602
AOR of mortality	–	–	0.51(0.27–1.04)	0.067

AOR, adjusted odds of ratio; CAD, coronary artery disease; CHF, congestive heart failure; CI, confidence interval; CVA, cerebral vascular accident; DM, diabetes mellitus; EZ-ALBI, easy albumin-bilirubin; ESRD, end-stage renal disease; GCS, Glasgow Coma Scale; HTN, hypertension; IQR, interquartile range; ISS, injury severity score; LOS, length of stay; OR, odds ratio; SD, standard deviation.

### Analysis of the ROC curve

**Figure 2:**
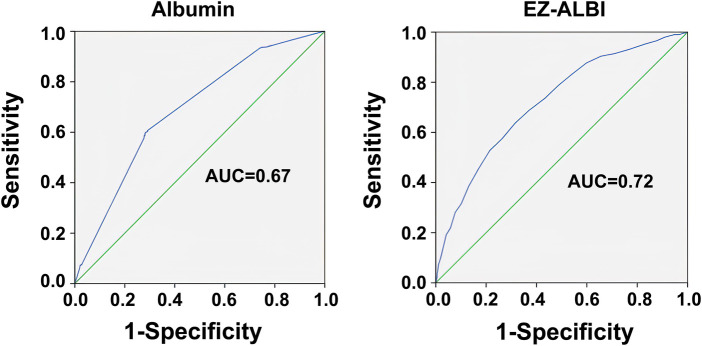
Receiver operating characteristic curves and area under the curve (AUC) of the EZ-ALBI score for predicting the mortality of the adult trauma patients by all trauma.

Based on the ROC analysis, the optimal albumin level was determined to be 3.33 g/dl, with a sensitivity of 0.600 and a specificity of 0.616 (Figure 2). The optimal EZ-ALBI score was determined to be −28.5, with a sensitivity of 0.637 and a specificity of 0.685 (Figure 2). Albumin alone and EZ-ALBI had AUCs of 0.67 and 0.72, respectively, as shown in Figure 2. The ability of EZ-ALBI alone to predict patient mortality was moderately accurate and superior to that of albumin alone (*p* = 0.046).

### Analysis of the risk factors for mortality

Univariate analysis revealed that sex, age, the presence of EZ-ALBI score ≥ −28.5, the level of glucose, AST, ALT, BUN, Cr, the presence of an injury of AIS ≥ 3 in the head/neck, extremities, or external, the presence of CAD, ESRD, or liver cirrhosis, the GCS score, and the ISS were significant risk factors for mortality in the study population (Table 3). Multivariate logistic regression analysis of these risk factors revealed that the presence of EZ-ALBI score ≥ −28.5 (OR, 2.31; 95% CI, 1.63–3.28; *p* < 0.001) was an independent risk factor for mortality. Additionally, age (OR, 1.02; 95% CI, 1.01–1.04; *p* < 0.001), the glucose level (OR, 1.23; 95% CI, 1.05–1.45 *p* = 0.011), injury to the external body region (OR, 9.48; 95% CI, 1.76–50.96 *p* = 0.009), liver cirrhosis (OR, 2.87; 95% CI, 1.42–5.78; *p* = 0.003), GCS (OR, 0.86; 95% CI, 0.83–0.90; *p* < 0.001), and ISS (OR, 1.05; 95% CI, 1.03–1.07; *p* < 0.001) were significant independent risk factors for mortality in these patients.

**Table 3:** Univariate and multivariate analysis of the risk factors for mortality of the patients.

Mortality	Univariate analysis			Multivariable analysis		
	OR	95%CI	*P*	OR	95%CI	*P*
Male, yes	1.52	(1.18–1.98)	0.001	1.38	(0.95–2.00)	0.095
Age, year	1.01	(1.00–1.02)	0.001	1.02	(1.01–1.04)	<0.001
EZ-ALBI ≥ −28.5, yes	3.87	(3.02–4.97)	<0.001	2.31	(1.63–3.28)	<0.001
Glucose, mg/dl	1.53	(1.35–1.74)	<0.001	1.23	(1.05–1.45)	0.011
AST, U/L	1.06	(1.02–1.10)	0.001	1.03	(0.97–1.09)	0.374
ALT, U/L	1.08	(1.01–1.15)	0.025	1.03	(0.91–1.16)	0.646
BUN, mg/dl	1.02	(1.01–1.02)	<0.001	1.01	(0.99–1.02)	0.268
Cr, mg/dl	1.14	(1.08–1.19)	<0.001	1.03	(0.95–1.13)	0.452
Head/neck (AIS ≥ 3), yes	5.25	(4.01–6.87)	<0.001	1.15	(0.73–183)	0.552
Extremities (AIS ≥ 3), yes	0.58	(0.43–0.78)	<0.001	0.63	(0.39–1.00)	0.051
External (AIS ≥ 3), yes	6.46	(2.15–19.40)	0.001	9.48	(1.76–50.96)	0.009
CAD, yes	1.87	(1.25–2.78)	0.002	1.48	(0.85–2.59)	0.168
ESRD, yes	3.17	(2.02–4.97)	<0.001	2.11	(0.88–5.10)	0.096
Liver cirrhosis, yes	2.26	(1.42–3.59)	0.001	2.87	(1.42–5.78)	0.003
GCS	0.79	(0.77–0.81)	<0.001	0.86	(0.83–0.90)	<0.001
ISS	1.09	(1.07–1.10)	<0.001	1.05	(1.03–1.07)	<0.001

AIS, abbreviated injury scale; ALT, alanine aminotransferase; AST, aspartate aminotransferase; BUN, blood urea nitrogen; CAD, coronary artery disease; CI, confidence interval; Cr, creatinine; EZ-ALBI, easy albumin-bilirubin; ESRD, end-stage renal disease; GCS, Glasgow Coma Scale; ISS, injury severity score; OR, odds ratio.

### The outcomes of patients with EZ-ALBI scores ≥ −28.5 vs. those with EZ-ALBI scores < −28.5

There was no significant difference in sex between patients with an EZ-ALBI score ≥ −28.5 and patients with an EZ-ALBI score < −28.5 (Table 4). Patients with an EZ-ALBI score ≥ −28.5 were significantly older than those with an EZ-ALBI score < −28.5 (*p* < 0.001). A significantly higher rate of an injury of AIS ≥ 3 in head/neck, thoracic, abdomen, and extremities body regions was found in patients with an EZ-ALBI score ≥ −28.5 compared to those with EZ-ALBI scores < −28.5. A significantly lower rate of pre-existing CVA, but no other comorbidities, was found in patients with an EZ-ALBI score ≥ −28.5 compared to those with EZ-ALBI scores < −28.5. Patients with an EZ-ALBI score ≥ −28.5 presented with a significantly lower GCS but a higher ISS than those with an EZ-ALBI score < −28.5 (GCS: 15 ([9–15]) vs. 15 ([14–15]), *p* < 0.001; ISS: 16 ([9–25]) vs. 9 ([8–18]), *p* < 0.001). Patients with an EZ-ALBI score ≥ −28.5 presented with a significantly higher mortality rate than patients with an EZ-ALBI score < −28.5 (15.1% vs. 4.4%, *p* < 0.001). Under the control of age, pre-existing CVA, GCS, and ISS, patients with an EZ-ALBI score ≥ −28.5 still presented with a significantly higher adjusted mortality rate than patients with an EZ-ALBI score < −28.5 (AOR, 2.47; 95% CI: 1.90–3.22, *p* = 0.001). Patients with an EZ-ALBI score ≥ −28.5 had significantly shorter hospitalization periods than those with an EZ-ALBI score < −28.5 (23.8 vs. 14.1 days, *p* = 0.001).

**Table 4:** Comparison of the injury, characteristics, and outcomes of patients with an EZ-ALBI score ≥ −28.5 vs. those with an EZ-ALBI score < −28.5.

Variables	EZ-ALBI≥ −28.5 *n* = 1,241	EZ-ALBI < −28.5 *n* = 2,396	OR (95%CI)	*p*
Gender				0.358
Male, *n* (%)	771 (62.1)	1,451 (60.6)	1.07 (0.93–1.23)	
Female, *n* (%)	470 (37.9)	945 (39.4)	0.94 (0.81–1.08)	
Age, years (SD)	59.2 ± 19.3	56.0 ± 19.5	–	<0.001
Trauma regions (AIS ≥ 3)				
Head/neck, *n* (%)	526 (42.4)	836 (34.9)	1.37 (1.19–1.58)	<0.001
Face, *n* (%)	12 (1.0)	11 (0.5)	2.12 (0.93–4.81)	0.067
Thoracic, *n* (%)	316 (25.5)	439 (18.3)	1.52 (1.29–1.80)	<0.001
Abdomen, *n* (%)	210 (16.9)	200 (8.3)	2.24 (1.82–2.75)	<0.001
Extremities, *n* (%)	471 (38.0)	592 (24.7)	1.86 (1.61–2.16)	<0.001
External, *n* (%)	8 (0.6)	6 (0.3)	2.58 (0.90–7.47)	0.069
Comorbidities				
CVA, *n* (%)	49 (3.9)	142 (5.9)	0.65 (0.47–0.91)	0.011
HTN, *n* (%)	434 (35.0)	814 (34.0)	1.05 (0.91–1.21)	0.548
CAD, *n* (%)	84 (6.4)	147 (6.1)	1.11 (0.84–1.47)	0.458
CHF, *n* (%)	16 (1.3)	24 (1.0)	1.29 (0.68–2.44)	0.430
DM, *n* (%)	261 (21.0)	455 (19.0)	1.14 (0.96–1.35)	0.142
ESRD, *n* (%)	43 (3.5)	83 (3.5)	1.00 (0.69–1.46)	0.999
GCS, median (IQR)	15 (9–15)	15 (14–15)	–	<0.001
ISS, median (IQR)	16 (9–25)	9 (8–18)	–	<0.001
1–15, *n* (%)	498 (40.1)	1,475 (61.6)	0.42 (0.36–0.48)	<0.001
16–24, *n* (%)	362 (29.2)	571 (23.8)	1.32 (1.13–1.54)	<0.001
≥25, *n* (%)	381 (30.7)	350 (14.6)	2.59 (2.20–3.06)	<0.001
Mortality, *n* (%)	187 (15.1)	105 (4.4)	3.87 (3.02–4.97)	<0.001
Mortality AOR	–	–	2.47 (1.90–3.22)	<0.001
Hospital LOS, days (SD)	23.8 ± 18.8	14.1 ± 14.0	–	0.001

AIS: Abbreviated Injury Scale; AOR, adjusted odds ratio; CAD, coronary artery disease; CHF, congestive heart failure; CI, confidence interval; CVA, cerebral vascular accident; DM, diabetes mellitus; ESRD, end-stage renal disease; EZ-ALBI, easy albumin-bilirubin; GCS, Glasgow Coma Scale; HTN, hypertension; IQR, interquartile range; ISS, injury severity score; LOS, length of stay; OR, odds ratio; SD, standard deviation. The AOR of mortality was calculated by adjusting for age, pre-existing CVA, GCS, and ISS.

### The outcomes of propensity score-matched cohort of patients with EZ-ALBI scores ≥ −28.5 vs. those with EZ-ALBI scores < −28.5

For patients with or without EZ-ALBI scores* *≥* *−28.5, a propensity score-matched patient cohort of 1:1 (Table 5) was established to reduce the influence of confounding factors related to the patients’ baseline characteristics of trauma mechanisms on outcome assessments. The propensity score-matched patient populations, comprising 1,236 pairings, exhibited no statistically significant variations in terms of trauma mechanisms, including traffic accidents, fall, strike by/against objects, suicide, and electric injury. Patients with an EZ-ALBI score* *≥* *−28.5 presented with a significantly higher mortality (OR, 4.12; 95% CI, 2.99–5.67, *p *<* *0.001) and longer LOS in the hospital (23.8 days vs. 14.8 days, *p *<* *0.001) than those with an EZ-ALBI score* *<* *−28.5.

**Table 5:** Comparison of outcomes of propensity score-matched cohort of patients with an EZ-ALBI score ≥ −28.5 vs. those with an EZ-ALBI score < −28.5.

Propensity Score-matched Patient Cohort					
	EZ-ALBI		OR (95% CI)	*P*	SD
	≥−28.5 n = 1,236	<−28.5 n = 1,236			
Traffic accident, *n* (%)	702 (56.8)	702 (56.8)	1.00 (0.85–1.17)	1.000	0.00%
Fall, *n* (%)	450 (36.4)	450 (36.4)	1.00 (0.85–1.18)	1.000	0.00%
Strike by/against, *n* (%)	59 (4.7)	59 (4.7)	1.00 (0.68–1.46)	1.000	0.00%
Suicide, *n* (%)	22 (1.8)	22 (1.8)	1.00 (0.55–1.82)	1.000	0.00%
Electric injury, *n* (%)	3 (0.2)	3 (0.2)	1.00 (0.20–4.96)	1.000	0.00%
Outcomes					
Mortality	186 (15.0)	51 (4.1)	4.12 (2.99–5.67)	<0.001	–
Hospital LOS, days	23.8 ± 18.8	14.8 ± 14.8	–	<0.001	–

CI, confidence interval; EZ-ALBI, easy albumin-bilirubin; LOS, length of stay; OR, odds ratio; SD, standardized difference.

## Discussion

In this study, patients who died were significantly associated with a higher EZ-ALBI score than those who survived, and those with an EZ-ALBI score ≥ −28.5 presented with a 2.47-fold adjusted mortality rate compared to patients with an EZ-ALBI score < −28.5. The analysis in a propensity score-matched pair cohort of 1,236 patients, which was developed to reduce baseline disparities in trauma mechanisms, also showed that patients with an EZ-ALBI score ≥ −28.5 had a 4.12 times higher mortality rate compared to patients with an EZ-ALBI score < −28.5. The results revealed that the EZ-ALBI score was a significant independent risk factor for mortality in adult trauma patients due to all trauma causes and presented with a significant better predictive power for mortality than the use of albumin alone. Therefore, EZ-ALBI may serve as a valuable tool to stratify the mortality risk of adult trauma patients.

The severity of liver dysfunction is often estimated using the MELD score or CTP classification. MELD is a continuous score derived from the calculation of serum creatinine and bilirubin levels and the international normalized PT ratio (55–57). However, MELD has been widely adopted for end-stage cirrhotic patients awaiting liver transplantation (2) and is specifically designed for patients with end-stage cirrhosis (58–60). The application of the MELD score in patients with less severe liver dysfunction has been criticized (4). In addition, the CTP classification system incorporates five different factors, including serum levels of total bilirubin, albumin, and prothrombin time, and two clinical symptom indicators, ascites and hepatic encephalopathy (3). It has been argued that the variable of ascites is intercorrelated with albumin, whereas it is difficult to subjectively assess and consistently score ascites and hepatic encephalopathy among different investigators (61); and the CTP score is limited by the arbitrary determination of cutoff values of objective laboratory variables with equal weighting of five parameters (62). Moreover, a literature review revealed that there were more than 30 versions of the CTP classification, making it difficult to achieve consistent scoring (63). Hence, the potential superiority of EZ-ALBI over CTP as a prognostic indicator for death in trauma patients is an intriguing subject that warrants additional research and exploration.

The EZ-ALBI score is a combination of two indicators, total bilirubin and albumin, which include both metabolic function (total bilirubin) and synthesis function (albumin) of the liver (64). Albumin and bilirubin levels are also frequently measured as part of the assessment of liver function and general health when conducting clinical practice. Increased serum bilirubin concentrations frequently indicate variable degrees of liver failure, serving as a predictor of liver performance in many prognostic models such as the Acute Physiology and Chronic Health Evaluation (APACHE) score (65), the Sequential Organ Failure Assessment (SOFA) score (66), Simplified Acute Physiology Score (SAPS II) (67), Logistic Organ Dysfunction Score (LODS) (68), and Multiple Organ Dysfunction Score (MODS) (69). Around 40 percent of critically ill patients have elevated bilirubin levels in the blood, which is associated with increased mortality and adverse outcomes (70). In addition, the decreased level of albumin, which is synthesized in the liver, suggests dysfunction in liver synthesis and malnutrition. Hypoalbuminemia may indicate malnutrition or inflammation, both of which are common in hospitalized patients (71). Inadequate albumin levels may lead to fluid imbalances, potentially producing edema, interfering with heart function, and increasing characteristics associated with poorer outcomes in trauma patients (72–75). Furthermore, albumin levels may indicate the degree of damage and total physiological stress, acting as a predictor of complications, prolonged ICU admission, and higher mortality risk (71). However, in contrast to bilirubin, albumin levels were not generally regarded as a principal variable in the majority of intensive care unit prediction models. Notably, these two groups of patients did not present significant differences in the level of albumin, total bilirubin, or the derived value of EZ-ALBI. The mortality and adjusted mortality corrected by age, incidences of HTN and DM, and ISS between the patients with and without major liver injuries did not present significant differences. It should be recognized that the liver dysfunction is not the only way for albumin and bilirubin to be changed by trauma. Although the mechanism underlying the prognostic impact of albumin and bilirubin remains undetermined, the ALBI approach based on laboratory data avoids interobserver variation and is superior to CTP in identifying patients with distinct prognostic subgroups within CTP (65). With easy calculation and assessment, EZ-ALBI may serve as a useful marker to help identify adult trauma patients with a high mortality risk.

This study has some limitations. First, there may have been a selection bias due to the retrospective design of this study. Second, management, such as damage control, blood transfusion, resuscitation, and surgical interventions, could have led to different outcomes in the study population; Furthermore, the physiology and nutritional condition, the laboratory data presented in the emergency room, the trauma mechanisms, and the injured regions can influence the patients' survival. All of these factors may introduce bias into the relationship assessment with the mortality outcome; however, we can only assume that the outcomes of these methods were uniform across the study population. Third, this study evaluated only in-hospital mortality and not the death declared upon arrival at the emergency room or long-term mortality; therefore, a selection bias may exist regarding comparing the outcomes. In addition, the exclusion of patients due to lack of bilirubin and albumin data resulted in the exclusion of a vast majority of trauma patients and may have resulted in selection bias. Fourth, this study included trauma patients due to all trauma causes and did not specify or exclude patients with liver injury. The impact of the liver injury on the application of EZ-ALBI in patients with trauma deserves further investigation. Finally, the study population was limited to a single urban trauma center; therefore, the generalizability of the results to other regions may be limited.

## Conclusion

This study revealed that the EZ-ALBI score was a significant independent risk factor for mortality and can serve as a valuable tool for stratifying mortality risk in adult trauma patients by all trauma causes.

## Data Availability

The original contributions presented in the study are included in the article/Supplementary Material, further inquiries can be directed to the corresponding author.
